# A novel method for γ photons depth of interaction discrimination on monolithic LYSO crystals for brain PET/MRI

**DOI:** 10.1186/2197-7364-2-S1-A11

**Published:** 2015-05-18

**Authors:** Roberto Pani, Marco Bettiol, Enrico Preziosi, Christian Borrazzo, Rosanna Pellegrini, Antonio Gonzalez, Pablo Conde, Maria Nerina Cinti, Andrea Fabbri, Elisabetta Di Castro, Stan Majewski

**Affiliations:** Department of Molecular Medicine, Sapienza University of Rome, Italy; Morphofunctional Sciences - Biophysics, Doctorate School of Biology and Molecular Medicine – Saimlal Department, Sapienza University of Rome, Italy; Post Graduate School of Medical Physics, Sapienza University of Rome, Rome, Italy; Institute for Instrumentation in Molecular Imaging, I3M-CSIC, Valencia, Spain; Department of Radiological Sciences, Oncology and Pathological, Sapienza University of Rome, Rome, Italy; West Virginia University, Morgantown, West Virginia USA

The MindView European Project pursues the development of a high efficiency and high resolution brain dedicated PET detector, simultaneously working with a Magnetic Resonance Imaging (MRI) system. Since the PET scanner is based on a small diameter ring and on thick monolithic scintillation crystals to assess high efficiency, the parallax error related to off-center positron annihilation is a critical issue. The Depth of Interaction (DoI) discrimination can reduce the blurring due to this phenomenon. In this work, we propose a novel DoI estimator, based on the ratio of the integral of scintillation light distribution to its maximum (named N/I). In a preliminary way, by means of Monte Carlo simulation, we have validated the correlation between this parameter and the DoI. Furthermore, we have experimentally tested the capability of such DoI estimator on a monolithic 20 mm-thick LYSO crystal optically coupled to a 12x12 silicon photomultipliers (SiPMs) array. Thanks to the proposed method, it is possible to select interaction events coming from different depths of the crystal. The DoI discrimination capability has been confirmed by using a collimated slanted pencil-beam: the proposed estimator allows to produce different images coming from events belonging to different depths of the crystal. From the experimental results a DoI discrimination resolution ranging from 4 mm to 6 mm has been obtained. The proposed method is expected to reduce the parallax error and, consequently, the width of lines of response coming from off- center positron annihilation of about 70% respect to the method without DoI discrimination.

